# Retraction: MNK1-Induced eIF-4E Phosphorylation in Myeloma Cells: A Pathway Mediating IL-6-Induced Expansion and Expression of Genes Involved in Metabolic and Proteotoxic Responses

**DOI:** 10.1371/journal.pone.0291491

**Published:** 2023-09-08

**Authors:** The PLOS ONE Editors

Following the publication of this article [1], concerns were raised regarding results presented in Fig 1. Specifically,

- The Fig 1A OPM-2 actin panel and the Fig 1C PT#4 actin panel appear similar when horizontally stretched despite representing different experimental conditions.
- Lanes 2–4 of the Fig 1A ANBL6/U266/8226 actin panel appear similar to the Fig 3A shRNA MNK2 GAPDH panel.
- The Fig 1C PT#2 P-MNK and T-MNK panels of [1] appear similar to the Figure 1B OPM-2 FKHD-P and FKHD-T panels of [2] respectively.
- In Fig 1D, the MNK1 panel in the MNK2 immunoprecipitation blot has no detectable signal nor does the panel present a positive control to confirm a successful blot.

The authors did not provide the raw data underlying the published results for editorial review. In the absence of the raw data required to support the published results PLOS is unable to resolve the above issues.

In light of the concerns affecting multiple figure panels that question the validity and reliability of these data, the *PLOS ONE* Editors retract this article.

CB and JG agreed with the retraction. PF did not agree with the retraction. YS, BH, YY, and AL either did not respond directly or could not be reached.
